# Beyond emotional intelligence ability: the power of supervisor–subordinate alignment in perceptions of supervisor emotionally intelligent behavior

**DOI:** 10.3389/fpsyg.2026.1637168

**Published:** 2026-03-03

**Authors:** Zehavit Levitats, Alisha Gupta, Alexander S. McKay, Zorana Ivcevic

**Affiliations:** 1 Department of Political Studies, Bar-Ilan University, Ramat Gan, Israel; 2 Department of Economics, Business, and Accounting, Randolph-Macon College, Ashland, VA, United States; 3 Human Resources Research Organization, Alexandria, VA, United States; 4 Yale Center for Emotional Intelligence, Yale University, New Haven, CT, United States

**Keywords:** emotional intelligence, emotionally intelligent behavior, engagement, burnout, job demands-resources model, self-other agreement theory

## Abstract

**Introduction:**

Emotional intelligence (EI) is widely recognized as critical to supervisory effectiveness. However, because EI is enacted and interpreted within relationships, it should be studied not only as individual ability but also as behavior and perception. This study examines how alignment or misalignment between supervisors’ and subordinates’ perceptions of supervisor emotionally intelligent behavior (EIB) relates to subordinate well-being, above and beyond supervisors’ actual EI ability.

**Methods:**

Data were collected from 202 supervisors and 2,055 subordinates across five hospitals, a high-stakes, emotionally charged environment particularly valuable for studying EI-related dynamics. We measured supervisor ability EI using the MSCEIT and assessed perceptions of supervisor EIB from both supervisors and subordinates. Multilevel polynomial regression was used to analyze perceptual alignment effects on subordinate engagement and burnout.

**Results:**

Controlling for supervisor ability EI, perceptual alignment on supervisor EIB predicted significantly higher subordinate engagement and lower burnout. Conversely, misalignment, particularly supervisor overestimation of their EIB, related to poorer subordinate outcomes.

**Discussion:**

These findings highlight the relational and interpretive nature of EIB in management, demonstrating that how supervisors’ emotional intelligence is perceived, and whether those perceptions align, matters for subordinate well-being.

## Introduction

Emotional intelligence (EI), the ability to monitor, discriminate between, and use one’s own and others’ emotions to guide their and others’ cognitions and behavior (Côté, 2014; Mayer and Salovey, 1997; Mayer et al., 2016), has gained significant traction among practitioners as a predictor of effective leadership (Elfenbein et al., 2015; Ivcevic et al., 2021; Saha et al., 2023). Yet, its relevance to supervisor effectiveness continues to be debated within the academic community (Ashkanasy and Daus, 2005; Antonakis et al., 2009; Cherniss, 2010; Dasborough et al., 2021; Landy, 2005; Locke, 2005; Roberts et al., 2010). One reason for this ongoing debate is the fragmentation of the literature into distinct research streams, each focused on conceptually distinct attributes (Ashkanasy and Daus, 2005). Stream 1 defines EI as a set of mental abilities (i.e., capacities to solve emotion-related problems) and measures EI by using performance-based tests scored based on objective criteria (e.g., MSCEIT; Mayer et al., 2003). Stream 2 defines EI as perceptions of ability and uses self-report scales to assess it (making it akin to emotional self-efficacy; Schutte et al., 1998; Wong and Law, 2002). Stream 3, “mixed EI” defines it as a broad set of traits and social–emotional attributes (e.g., Goleman, 1998). This study focuses on emotionally intelligent behavior (EIB), actions that reflect the perceiving, using, understanding, and regulating of emotions, which are influenced by the ability and motivation for such behavior, as well as contextual-organizational factors (Levitats et al., 2025).

Barring a few exceptions, all of which are in non-managerial contexts (Brackett and Mayer, 2003; Brackett et al., 2006; Law et al., 2004), research on the role of supervisors’ EI in predicting subordinate work outcomes has typically focused on the second EI stream (i.e., self-reports) and relied on one rating source. Most research assesses how subjective perceptions of supervisor EI, reported by either subordinates or supervisors, relate to subordinate outcomes. This approach overlooks the possibility that there may be misalignment in these perceptions and that the degree of (mis)alignment between supervisors’ and subordinates’ perceptions with regard to supervisor EI may in itself influence subordinates’ experiences at work. Exploring this (mis)alignment is especially important in light of meta-analytic findings showing there is only moderate agreement between supervisors’ self-ratings and subordinate ratings of supervisors’ behavior (Fleenor et al., 2010; Lee and Carpenter, 2018), with modest correspondence observed specifically for EI (Elfenbein et al., 2015). This limited agreement raises fundamental questions about the social construction of supervisor EIB and its relationship to subordinate outcomes. More specifically, to what extent does the alignment between supervisors’ and subordinates’ perceptions of supervisor EIB matter, beyond the supervisor’s actual EI ability? How do congruent versus incongruent perceptions of supervisor EIB relate to subordinate outcomes?

This study addresses these gaps by incorporating both supervisors’ and subordinates’ perceptions of supervisor EIB. Levitats et al. (2025) have proposed that such behavior is based on one’s ability (emotion-related know how), motivation (efficacy to enact behavior and value ascribed to it), and contextual opportunities (supports and barriers). We compare supervisors’ perceptions of their own behavior and how their subordinates’ perceive it, assessing the degree of (mis)alignment between them, and its relationship with two critical subordinate well-being outcomes: work engagement and burnout. Work engagement refers to a positive, fulfilling work-related state characterized by vigor, dedication, and absorption in one’s work (Schaufeli et al., 2002). Burnout represents a state of physical, emotional, and mental exhaustion, resulting from long-term involvement in mental and emotional demands at work (Schaufeli and Greenglass, 2001). The two constructs are commonly regarded as the most indicative of well-being at work (see Galanakis and Tsitouri, 2022; Schaufeli, 2017; Zhou et al., 2024).

These well-being indicators are particularly fitting for this study. Because supervisors exert substantial influence over subordinate daily experiences (e.g., by setting expectations, coordinating tasks, and providing social and emotional support), subordinate well-being is highly responsive to the dynamics of this relationship (Breevaart and Bakker, 2018; Decuypere and Schaufeli, 2020; Heyns et al., 2022; Xu and Farris, 2024). Through emotion recognition, regulation, and response patterns, supervisors influence how subordinates interpret workplace events and manage emotional demands. This is especially relevant for work engagement and burnout because both indicators of work-based well-being directly capture subordinates’ emotional experiences at work (Karahan Kaplan et al., 2024).

We integrate two theoretical frameworks – Self-Other Agreement theory (SOA; Atwater and Yammarino, 1992) and Job Demands-Resources model (JD-R; Bakker and Demerouti, 2017, 2024). The JD-R model is the dominant framework for linking job characteristics to subordinate well-being in various occupational settings including healthcare (for reviews and meta-analyses see Galanakis and Tsitouri, 2022; Vargas-Benítez et al., 2023). The model posits that workplace characteristics function as either job demands (i.e., aspects requiring sustained effort and associated with costs, such as health impairment) or job resources (i.e., aspects that facilitate goal achievement, reduce demands, and stimulate growth). Self–Other Agreement theory (Atwater and Yammarino, 1992, 1997) holds that supervisors’ self-ratings should be compared with subordinates’ perceptions because these views often diverge, creating different patterns of agreement or discrepancy. These patterns, whether congruent or incongruent, carry distinctive implications, with alignment generally supporting more positive outcomes and misalignment producing varying effects depending on whether supervisors overestimate or underestimate their behavior. We draw on SOA theory (Atwater and Yammarino, 1992, 1997) to theorize that patterns of EIB perceptual (mis)alignment function as either resources or demands for subordinates.

Supervisor-subordinate aligned perceptions of supervisor EIB reflect reduced partner uncertainty, defined as the difficulty to reliably anticipate or interpret a partner’s attitudes and behaviors within interaction (Berger and Bradac, 1982; Knobloch and Solomon, 1999; Kreye, 2017). When both supervisor and subordinate share similar views of the supervisor’s EIB, whether high or low, they hold comparable expectations about the emotional tone, quality, and reliability of supervisory interactions, thereby minimizing ambiguity in the relationship. In contrast, misaligned perceptions heighten relational uncertainty by creating discrepant expectations about the supervisor’s emotional responsiveness and support. Consistent with evidence that uncertainty operates as a job demand that drains psychological resources and predicts emotional exhaustion, while reduced uncertainty serves as a job resource that supports well-being (Usman et al., 2023), this study conceptualizes partner uncertainty as a central psychosocial mechanism linking perceptual (mis)alignment in supervisor perceived EIB to subordinate well-being. From a methodological point of view, by factoring in supervisors’ EI ability (MSCEIT; Mayer et al., 2003) scores, we isolate the unique impact of perceptual (mis)alignment from supervisor’s actual EI ability and examine this isolated effect on subordinate well-being, advancing our understanding of how socially constructed perceptions of emotional competence shape subordinate experiences.

## Theoretical background

### Emotional intelligence

Emotional intelligence was coined to denote individual capacity to reason with and about emotions in relation to four interconnected abilities: (1) Perceiving emotions accurately in oneself and others through interpretation of clues such as facial expressions, body language, and tone; (2) Using emotions strategically to facilitate decision making and enhance problem-solving by optimizing mood-thinking relationships; (3) Understanding typical origins, evolution, and outcomes of emotions; and (4) Managing emotions effectively in oneself and others to achieve desired hedonic and instrumental outcomes (Mayer and Salovey, 1997). In addition to research on EI as a set of mental abilities applied to emotional subject matter, organizational behavior scholars studied how individuals perceive their emotional abilities. These self-perceptions are akin to emotional self-efficacy (Kirk et al., 2008; Wong and Law, 2002). Both EI ability and self-perceptions have been related to outcomes related to relationship effectiveness, performance, and well-being (Miao et al., 2016, 2017, 2018; O'Boyle et al., 2011; Sánchez-Álvarez et al., 2016).

Recent theory has pointed to the difference between individual potential and enacted behavior (Ivcevic et al., 2025; Levitats et al., 2025). This distinction is important because internal attributes such as ability and self-perceptions are not the most proximal influences on relevant outcomes in the organizational contexts. They are both attributes describing potential for particular behavior. The most direct influence on work outcomes is exerted by enacted behavior (Boyatzis, 2018; Ivcevic et al., 2025; Levitats et al., 2025). For this reason, the present research focuses on emotionally intelligent behavior (EIB) by assessing supervisor perceptions of their own actions and employee perceptions of how their supervisors act.

Supervisor EIB is related to employee experience of work (perceived ability to grow, greater experience of positive emotions) and their performance (e.g., creative behavior at work; Ivcevic et al., 2021). Supervisor EIB also mediates the relationship between contextual variables, such as EI-focused human resource practices and employee well-being-related outcomes (Levitats et al., 2022). The present study goes a step further and acknowledges that EIB is enacted in a social context. Supervisors interpret their own behavior and their subordinates perceive it, with similarities and differences between them having implications for relevant employee outcomes.

### Self–other agreement theory applied to EIB

Self-Other Agreement theory (Atwater and Yammarino, 1992, 1997) emphasizes the importance of comparing supervisors’ self-ratings with others’ perceptions, since supervisor self-ratings often diverge from subordinate ratings (Atwater et al., 2005). The theory delineates four patterns of supervisor-subordinate perceptions: Two congruence patterns: in-agreement good (high ratings from both self and other) and in-agreement poor (low ratings from both sources); and two incongruence patterns: supervisor underestimation (supervisors’ self-ratings lower than other-ratings) and supervisor overestimation (supervisors’ self-ratings higher than other-ratings) (Atwater et al., 2005). Agreement generally yields better outcomes (e.g., supervisor effectiveness, subordinate well-being) than disagreement, by fostering mutual understanding and expectations (Amundsen and Martinsen, 2014; Hasson et al., 2019). When perceptions diverge, over-raters show poorer outcomes as they fail to recognize and address others’ concerns, while under-raters demonstrate mixed outcomes due to their competence and drive for improvement (Atwater et al., 2005).

In the context of supervisors’ EIB, congruence can take one of two forms. In high–high cases, supervisors believe they effectively notice, interpret, and respond to subordinates’ emotions, and subordinates experience these behaviors similarly. In low–low cases, both parties recognize that the supervisor does not consistently display emotionally intelligent behaviors. Incongruence may occur as overestimation, when supervisors believe they acknowledge staff emotions, provide support, and handle sensitive situations well, while subordinates experience them as inattentive or dismissive of emotional cues. Alternatively, incongruence can arise when supervisors underestimate their EIB, rating themselves as less effective emotionally even though subordinates perceive them as empathetic, supportive, and skilled at managing emotional dynamics. To the best of our knowledge, the framework has not yet been applied to EI research, making the present study a novel extension of SOA theory into the EI domain.

### Job demands-resources model applied to EIB

The Job Demands-Resources (JD-R) model (Bakker and Demerouti, 2017, 2024) categorizes workplace characteristics into two elements: demands and resources. Job demands require ongoing exertion and create physiological and psychological strain, while job resources facilitate goals, minimize demands, and enable development through elements like supportive relationships (Demerouti et al., 2001). Demands and resources trigger a health-impairment process and a motivational process, respectively. Persistent demands lead to burnout by depleting energy through continuous coping, while resources foster engagement in two ways: intrinsically, by supporting personal development and growth, or extrinsically, by helping achieve work objectives (Bakker and Demerouti, 2017; Demerouti and Bakker, 2023). Beyond these distinct processes, the model explains how demands and resources interact. Resources can buffer the negative effects of demands on well-being, attenuating their energy-depleting impact when resources are abundant (Bakker et al., 2005; Shin and Hur, 2021a, 2021b). Additionally, challenging demands can amplify resources’ positive impact on engagement (Breevaart and Bakker, 2018; Tadić et al., 2015), making resources more motivating when work is complex.

Through the lens of the JD-R model (Bakker and Demerouti, 2007, 2024), supervisors’ EIB can function as a critical job resource. High-EI supervisors contribute to supportive work environments where subordinates are better equipped to manage emotional demands, access social support, and exercise autonomy (Bakker, 2015; Côté, 2007). Their ability to accurately interpret and respond to subordinate emotional cues (Côté, 2014, 2017) enables them to provide constructive feedback, resolve interpersonal challenges (Morrison, 2008), and guide subordinate growth and development (Clore et al., 1994; Schwarz, 2012). Moreover, supervisors who behave in emotionally intelligent ways foster engagement not only through strategic leadership behaviors, such as articulating vision and providing recognition, but also through interpersonal behaviors like team building and empowerment (Guillen and Florent-Treacy, 2011). By effectively managing emotional dynamics, they help subordinates cope with emotional challenges and promote positive affective states (Momeni, 2009), which can reduce burnout and enhance engagement. Consistent with these mechanisms, subordinates who perceived their managers as high in EIB reported greater growth opportunities, more positive emotions, and higher creativity and innovation at work (Ivcevic et al., 2021).

Conversely, low managerial EIB may function as a job demand. When supervisors lack emotional awareness and regulation skills, subordinates are more likely to experience emotional strain, reduced trust (Dunn and Schweitzer, 2005), and depleted personal resources as they navigate difficult supervisory relationships (Levitats et al., 2019). Indeed, evidence suggests that subordinates’ own self-reported EI is positively associated with engagement only when they perceive their supervisors as demonstrating high EIB, highlighting the pivotal role of managerial EIB in shaping the emotional climate of their workplace (Levitats et al., 2019). Together, these findings position supervisor EIB as a key contextual factor that can either buffer against or exacerbate job stressors, depending on its presence or absence.

### Hypotheses development

**Figure 1:**
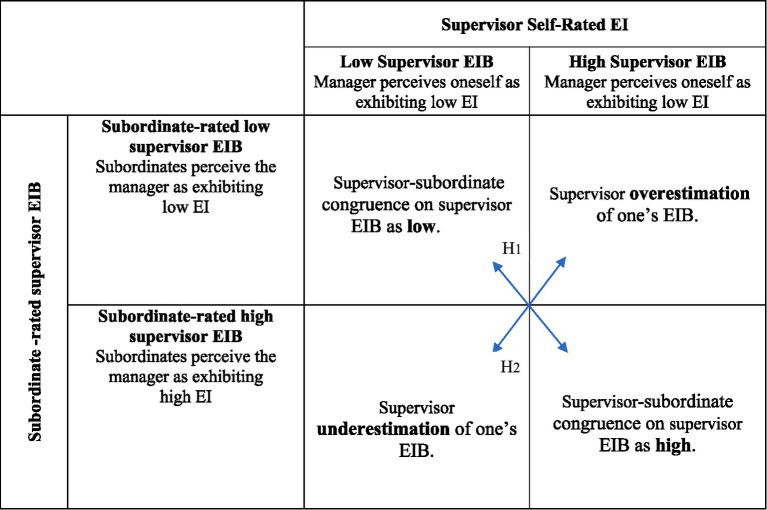
Matrix of supervisor-subordinate (in)congruence of supervisor EI. H1: Manager-subordinate congruence on manager EIB has a negative relationship with subordinate work-related burnout (*H1a*) and a positive relationship with subordinate job engagement (*H1b*). H2: Manager-subordinate incongruence on manager EIB has a positive relationship with subordinate work-related burnout (*H2a*) and a negative relationship with subordinate job engagement (*H2b*), such that burnout will be lower and job engagement will be higher when managers underestimate their EI compared to when managers overestimate it.

**Figure 2:**
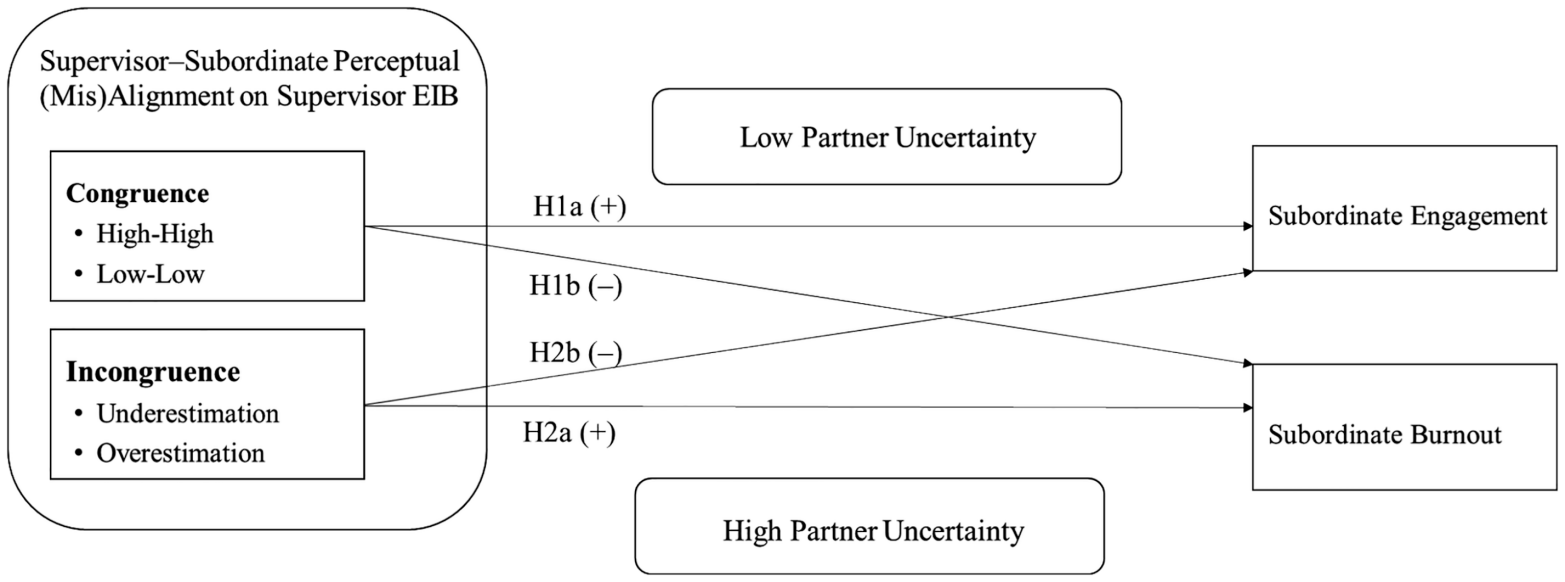
Conceptual model of supervisor–subordinate perceptual (mis)alignment in supervisor emotionally intelligent behavior and subordinate wellbeing.

Beyond the theoretical role EI ability plays in subordinate well-being, we draw on SOA theory and the JD-R model (Bakker and Demerouti, 2017, 2024), to propose that subordinate engagement and burnout vary across the various types of (in)congruence between supervisor and subordinate perceptions of supervisor EIB. The hypothesized differential effects of supervisor-subordinate congruence and incongruence in ratings of EI-relevant behavior are presented in Figures 1, 2 and discussed next.

### Supervisor–subordinate congruence on supervisor EIB

Congruence occurs when the supervisor’s self-ratings align with subordinates’ ratings. In the context of EIB, supervisor-subordinate congruence indicates shared perceptions of the supervisor’s behavior indicating the capacity to recognize, understand, and regulate one’s own emotions and those of others. Agreement on high supervisor EIB (i.e., in-agreement good) represents the upper end of the congruence continuum, whereas agreement on low EIB (i.e., in-agreement poor) represents its lower end.

SOA theory shows that mutual perceptions of the supervisor are positively associated with desirable subordinate outcomes (Fleenor et al., 2010). Applied here, supervisor-subordinate agreement on supervisor EIB should be related to subordinates’ well-being. We propose that the match itself is the critical mechanism, as perceptual congruence reflects reduced partner uncertainty as well as subordinates’ increased sense of predictability and efficacy in dealing with the environment. Subordinates’ enhanced perceived efficacy may serve as a job resource that fosters their engagement and reduces burnout, by allowing them to develop realistic expectations and coping strategies. This logic is consistent with the JD-R model (Bakker and Demerouti, 2017, 2024), which identifies reduced uncertainty as a job resource and heightened uncertainty as a job demand (Bakker and Demerouti, 2017, 2024; Usman et al., 2023), thereby enhancing engagement and reducing burnout. Likewise, these shared perceptions may initiate a resource gain spiral (Hobfoll et al., 2018; Westman et al., 2004) where supervisor EIB generates additional resources and enhances well-being. The above reasoning leads us to hypothesize:

*H1:* Supervisor-subordinate congruence on supervisor EIB has a negative relationship with subordinate work-related burnout *(H1a)* and a positive relationship with subordinate job engagement *(H1b)*.

### Supervisor–subordinate incongruence on supervisor EIB

According to SOA theory (Atwater and Yammarino, 1992, 1997) incongruence occurs when supervisors’ self-ratings diverge from subordinate ratings of the supervisor’s behavior, manifesting in two forms: underestimation and overestimation. Subordinates experience distinct outcomes when working under managers who either overestimate or underestimate themselves, with overestimation creating more challenging work conditions (Tsai et al., 2022). Research on supervisor-subordinate perceptual gaps shows that subordinate outcomes are particularly negative when team members identify a problem that their supervisor fails to recognize, compared to situations where supervisors perceive problems that teams do not (Bashshur et al., 2011; Hasson et al., 2019).

Through the lens of the JD-R model (JD-R; Bakker and Demerouti, 2017, 2024), subordinates working with EIB-overestimating supervisors are likely to face both increased demands and depleted resources. Overestimating supervisors are characterized by inflated self-perception and low awareness (Sheldon et al., 2014). When supervisors overestimate their EIB, the gap between expected and delivered EI-related behavior creates repeated unmet expectations. Over time, this discrepancy may lead subordinates to experience partner uncertainty and to feel disillusioned or disappointed. Consequently, subordinates must expand additional emotional and cognitive resources managing interactions where emotion-relevant behavior is expected but not delivered. This combination of depleted emotional resources and heightened demands typically results in increased burnout and decreased work engagement (Bakker and Demerouti, 2017, 2024), as subordinates exhaust personal resources attempting to fill the emotional gap left by their supervisors.

Subordinates working with underestimating supervisors are also likely to experience partner uncertainty, which is expected to be associated with higher burnout and lower engagement. However, their outcomes are likely to be more favorable than in the case of overestimating supervisors, because under-estimators tend to generate job resources that may buffer the negative effects of uncertainty. Underestimating supervisors are typically more self-critical and therefore more motivated to improve (Tekleab et al., 2008), leading them to invest greater effort in exercising behaviors that utilize their emotional abilities. As a result, they may exercise EIB, such as active feedback-seeking, careful attention to emotional cues (Ashkanasy and Humphrey, 2011), and effective emotion regulation (Liu and Liu, 2013). These resource-enhancing behavior helps subordinates cope with work demands, reducing burnout (Chen and Chen, 2018) and fostering positive emotional states that promote engagement (Ouweneel et al., 2012). Thus, although partner uncertainty is present, subordinates of underestimating supervisors may experience better well-being outcomes than those whose supervisors overestimate their EIB. This reasoning leads to the following hypotheses:

*H2:* Supervisor-subordinate incongruence on supervisor EIB has a negative relationship with subordinate work-related burnout *(H2a)* and a positive relationship with subordinate job engagement *(H2b)*, such that burnout will be lower and job engagement will be higher when supervisors underestimate their EI compared to when supervisors overestimate it.

## Methods

### Participants

Following ethical approval, the survey was administered to all staff, physicians, and residents (*N* = 16,166; *n* = 871 supervisors and *n* = 15,295 subordinates) working in five hospitals in Ontario, Canada. Hospitals were selected as the research setting because they offer a uniquely valuable context for studying EIB alignment. These environments are emotionally intense, with staff routinely encountering life-and-death situations, patient suffering, and distressed families, as well as regulatory and funding challenges—factors that can impact subordinate well-being (Grandey et al., 2012). A total of 6,272 people (492 supervisors and 5,780 subordinates) provided informed consent and responded to the survey, with average response rate across hospitals of 38.80% (56.49% for supervisors and 37.79% for subordinates). Total response rates were similar across hospitals (between 36.13 and 52.35%). In the above counts, supervisors were considered those with at least one direct report, and subordinates had zero direct reports. Notably, the analyses controlled for middle managers who are included as both subordinates and supervisors. The supervisor and subordinate samples are mutually exclusive. The subordinate count including both non-managing employees and middle managers is 6,187. The distinction of subordinate /supervisor levels are noted in the demographics and controlled for in the analyses.

Careless responders were screened for using three instructed response items (i.e., ‘select strongly agree for this item’; Huang et al., 2015). Following prior studies (e.g., Cho and Allen, 2012; Shockley et al., 2016), 45 supervisors (9.15%) and 896 subordinates (14.48%) who failed at least two of these checks were removed. Additionally, participants without matching supervisor-subordinate data (i.e., subordinates without a supervisor participating and supervisors without participating subordinates) were excluded as analyses required both ratings. Supervisors who did not complete the EI ability measure were excluded as well. The final sample included 202 supervisors and 2,055 subordinates.

For the final sample of supervisors, there were 129 females and 73 males. On average, supervisors were 48.18 years old (*SD* = 9.40) with 12.71 years of job tenure (*SD* = 10.44). One hundred eighty supervisors reported they were White/Caucasian, seven reported they were Asian, two reported they were African, and 13 reported they were Mixed/Other. On average, supervisors had 10.17 direct reports (*SD* = 11.12, Median = 6). One hundred seventy-five supervisors were in middle management positions (they were subordinates to another supervisor and had their own subordinates), whereas 27 were in senior management positions (they only had subordinates).

For the final sample of subordinates, there were 1,572 females and 483 males. On average, subordinates were 42.20 years old (*SD* = 11.32) with 11.40 years of job tenure (*SD* = 9.81). One thousand seven hundred one subordinates reported they were White/Caucasian, 110 reported they were Asian, 59 reported they were African, and 170 reported they were Mixed/Other (15 were missing). There were 1,854 participants who were solely subordinates and 201 who were middle managers.

### Measures

#### Self-rated and subordinate-rated supervisor EIB

Self- and subordinate-perceptions of supervisor EIB were measured with Ivcevic et al.'s (2021) 11-item scale using a six-point scale (strongly disagree-strongly agree). This self-report measure corresponds to the four ability EI branches: (a) perceiving and expressing emotions (three items), (b) using emotions (four items), (c) understanding emotions (two items), and (d) regulating emotions (two items). Although the items assess all four EI branches, the scale is unidimensional. When completing the scale, supervisors and subordinates provided self-ratings, and subordinates rated their immediate supervisor. A self-report sample item is ‘If someone is feeling upset about a decision, I will notice.’ For subordinate-report of supervisor EIB, ‘I am’ was changed to ‘My supervisor is.’ Cronbach’s *α*: subordinate self-report = 0.84, supervisor self-report = 0.84, and subordinate-report of supervisor EIB = 0.97.

#### Subordinate work-related burnout

The seven items from the work-related component of the Copenhagen Burnout Index (Kristensen et al., 2005) were used. Subordinates self-reported burnout using a six-point scale (strongly disagree-strongly agree). A sample item is ‘I feel worn out at the end of the working day’ (Cronbach’s α = 0.91).

#### Subordinate job engagement

Job engagement was measured with six of the 18 items from Rich et al. (2010). Best-practices for creating and using ‘new’ scales (Heggestad et al., 2019) were followed. The two items with the strongest factor loadings from each of the three engagement subscales (physical, emotional, cognitive) were chosen. A sample item is ‘I devote a lot of energy to my job’ (Cronbach’s α = 0.74). To determine if the shorter scale was a valid measure reflective of the original scale, both the short and full forms were administered via the CloudResearch research panel to a separate sample of full-time, working adults in the U.S. After removing careless responders, there were 313 people (Age: *M* = 39.51, SD = 10.66; Gender identity: 191 Males, 119 Females, 1 Non-binary/third gender, and 2 preferred not to answer; Job Tenure: *M* = 8.35, SD = 7.10; Education: most participants [46%] had a four-year post-secondary degree). The relationship between the short-form (α = 0.83) and full-form (α = 0.95) was 0.88.

#### Control variables

Multiple variables were controlled to rule out alternative explanations. A measure of EI ability was included to help determine whether supervisor-subordinate (in)congruence accounts for subordinate well-being, beyond the effect of supervisor EI ability. Supervisors completed the MSCEIT v2.0 developed by Mayer et al. (2003). It is a performance measure that assesses individuals’ proficiency in solving emotion-laden problems across all four EI branches. The test contains 141 items and answers are scored based on emotion science expert judgments. To ensure there was a match in breadth between ability and self−/other-reported measures, total EI ability scores were used. The MSCEIT User’s Manual reports split-half reliability coefficient of 0.91 for the total score (Mayer et al., 2002).

Additional controls were included: First, four dummy variables were created for the five hospitals, with the largest hospital serving as the referent. Supervisor and subordinate demographics (gender identity, age, tenure) were controlled for, as they correlate with EI-relevant measures (Cabello et al., 2016) and the outcomes (McKee et al., 2018). Third, supervisor and subordinate type (senior managers/middle managers for supervisors; employees/middle managers for subordinates) were controlled for. Finally, subordinate self-report EIB was controlled for, as self-reported EI-relevant variables relate to engagement (George et al., 2022) and burnout (see systematic review by Mérida-López and Extremera, 2017). In line with Bernerth and Aguinis's (2016) recommendations, all analyses were additionally conducted without control variables. The primary regression coefficients of interest remained statistically significant even when control variables were excluded, further supporting the study’s hypotheses.

## Data analysis

Because subordinates (Level 1) were nested under supervisors (Level 2), data was analyzed using multilevel modeling (Bliese and Hanges, 2004). All subordinate-reported variables are Level 1 and all supervisor-reported variables (e.g., demographics, self-reported EIB, and EI ability) are Level 2. Subordinate level variables should be clustered by supervisor because supervisors created a shared emotional and social context that influences their subordinates’ experience, thus, subordinates under the same supervisor are not independent of one another. Analytically, using traditional ordinary least squares (OLS) regression would violate the assumptions of independency and result in biased results. To justify using multilevel modeling, it was determined which level the majority of variance was at, using ICC(1) values (LeBreton and Senter, 2008). Most of the variability was at the subordinate-level: subordinate self-reported EIB = 96.50%, subordinate-reported supervisor EIB = 75.01%, subordinate job engagement = 94.64%, and subordinate burnout = 84.96. Thus, it was concluded that multilevel analyses are appropriate. In contrast, aggregating subordinate scores to Level 2 would obscure meaningful within-group variability in subordinate variables such as engagement and burnout, which are inherently individual-level experiences. Moreover, aggregation is misfitting as the central focus of this study is the congruence between each subordinate and their specific supervisor, not the congruence between an aggregated group average and the supervisor score. To conclude, multilevel modeling allows us to preserve the within-group information while analyzing cross-level effects of supervisor characteristics.

Prior to creating the polynomial terms for the analyses, supervisor self-report and subordinate-report of supervisor EIB were centered. Because we were specifically interested in interpreting the lines of congruence and incongruence of the response surface, it was important to ensure that the polynomial terms are centered using the same value (Humberg et al., 2020; Schönbrodt, 2016). This ensures that discrepancies from the central point in the response surface are comparable for both terms. Centering was done using the mean of the combined supervisor EIB variables. Grand-mean centering was also used for supervisor EI ability.

Hypotheses 1 and 2 were tested using multilevel polynomial regression (Nestler et al., 2019) and response survey analyses using the RSA R package v0.10.4 (Schönbrodt and Humberg, 2021) and multilevel RSA functions in R developed by Nestler et al. (2019). Polynomial regression allows researchers to test the (in)congruence, or (dis)agreement, between two independent variables on a dependent variable. It is an alternative to difference score approaches, which are often unreliable (Edwards and Parry, 1993). Multilevel polynomial regression is called for when the data spans multiple levels of analysis. After conducting these regression analyses, the regression coefficients are used to test hypotheses related to (in)congruence in scores and conduct response surface analyses in which a three-dimensional surface of the results is created (Tsai et al., 2022). These make it possible to evaluate the slope and curvature for both the line of congruence (i.e., agreement among supervisors and subordinates regarding supervisor EIB) and line of incongruence (i.e., disagreement among supervisors and subordinates). For polynomial regression, there are various considerations for test of statistical significance (Edwards and Parry, 1993). A thorough description of these considerations and the procedures used to align with them is provided in Supplementary Appendix 1.

To examine whether the results depend on supervisor EI ability, multilevel moderated polynomial regression was conducted (Nestler et al., 2019). This final model (Model 3) included all control variables, the five main polynomial terms, the moderator (supervisor EI ability), and the five moderated polynomial terms (the five main polynomial terms moderated by supervisor EI ability). Incremental significance for the entire block of moderated terms was examined – based on changes in model deviance scores from the model with control variables and polynomial terms. Additionally, *R*^2^ values were considered for tests of practical significance of each model.

## Results

Table 1 includes the means, standard deviations, and zero-order correlations of all variables in the study. The correlations for the level 2 variables are presented separately from level 1 variables. Notably, the relationship between supervisor self-report EIB, supervisor subordinate-report EIB, and supervisor EI ability were similar to those reported in prior research (Elfenbein et al., 2015). Of particular interest, supervisor self-reported EIB was positively, yet modestly, related to subordinate rating (*r* = 0.09, *p* < 0.001) and positively associated to supervisor EI ability (*r* = 0.24, *p* < 0.001).

**Table 1:** Correlations among study measures.

	*M*	*SD*	1	2	3	4	5	6	7	8	9	10	11	12	13	14	15	16	17
Supervisor level (*n* = 202)																			
1. Hospital (Dummy 1)	0.07	0.26																	
2. Hospital (Dummy 2)	0.05	0.23	−0.07																
3. Hospital (Dummy 3)	0.07	0.26	−0.08	−0.07															
4. Hospital (Dummy 4)	0.20	0.40	−0.14*	−0.12	−0.14*														
5. Supervisor Sex	1.36	0.48	−0.02	−0.09	−0.06	0.07													
6. Supervisor Age	48.18	9.40	0.01	0.12	0.02	0.05	−0.07												
7. Supervisor Tenure	12.71	10.44	−0.21**	−0.05	−0.09	−0.08	−0.18*	0.25***											
8. Supervisor Type	1.13	0.34	−0.06	0.61***	0.72***	−0.20**	−0.11	0.10	−0.12										
9. Supervisor S. R. EI	5.03	0.43	−0.05	−0.05	0.03	−0.11	−0.04	0.06	0.16*	0.02									
10. Supervisor EI Ability	100.77	14.83	−0.04	−0.10	0.03	0.05	−0.12	−0.01	0.00	−0.01	0.20**								
Subordinate level (*n* = 2,055)																			
1. Hospital (Dummy 1)	0.07	0.26																	
2. Hospital (Dummy 2)	0.02	0.14	−0.04																
3. Hospital (Dummy 3)	0.04	0.20	−0.06**	−0.03															
4. Hospital (Dummy 4)	0.13	0.33	−0.11***	−0.05*	−0.08***														
5. Supervisor Sex	1.31	0.46	0.02	−0.06**	−0.07**	0.17***													
6. Supervisor Age	47.25	9.25	−0.05*	0.14***	−0.01	0.06*	−0.19***												
7. Supervisor Tenure	15.78	11.17	−0.24***	−0.01	−0.07**	−0.13***	−0.24***	0.46***											
8. Supervisor Type	1.06	0.24	−0.06**	0.54***	0.81***	−0.10***	−0.10***	0.07**	−0.07**										
9. Subordinate Sex	1.24	0.42	−0.04	−0.03	−0.06**	0.08***	0.27***	−0.01	−0.01	−0.07**									
10. Subordinate Age	42.20	11.32	−0.02	0.04	0.02	0.07**	0.05*	0.09***	−0.01	0.05*	0.08***								
11. Subordinate Tenure	11.40	9.81	−0.13***	−0.03	−0.03	−0.06**	−0.06**	0.05*	0.11***	−0.05*	−0.02	0.61***							
12. Subordinate Type	1.10	0.30	0.00	−0.03	−0.07**	0.10***	0.09***	0.10***	−0.06**	−0.06*	0.10***	0.14***	0.05*						
13. Subordinate S. R. EIB	4.79	0.53	0.03	0.01	−0.02	0.05*	−0.01	0.02	−0.03	−0.01	−0.06**	0.02	−0.01	0.13***					
14. Supervisor F. R. EIB	4.15	1.23	0.00	0.03	−0.05*	0.02	0.04	−0.01	−0.07**	−0.02	0.03	0.04	−0.06**	0.15***	0.15***				
15. Supervisor S. R. EIB	5.03	0.41	−0.05*	−0.13***	−0.01	−0.11***	−0.04	0.12***	0.29***	−0.07***	0.01	0.00	0.00	0.11***	0.04	0.09***			
16. Supervisor EI Ability	101.50	15.57	−0.11***	−0.15***	−0.04	0.02	−0.12***	0.10***	0.18***	−0.11***	−0.08***	−0.03	0.02	0.00	−0.01	−0.10***	0.24***		
17. Subordinate Job Engagement	4.93	0.63	0.08***	−0.01	−0.01	−0.04	−0.03	0.10***	−0.02	−0.01	−0.04	0.08***	−0.02	0.14***	0.36***	0.19***	0.03	0.05*	
18. Subordinate Burnout	3.48	1.13	0.01	−0.06**	0.00	−0.01	−0.02	−0.10***	−0.06**	−0.04	−0.03	−0.05*	0.09***	−0.03	−0.06**	−0.35***	−0.01	0.06*	−0.17***

Supervisor/Subordinate Gender: 1 = Women, 2 = Men. Supervisor Type: 1 = Middle Manager (i.e., both a subordinate and supervisor), 2 = Senior Manager; Subordinate Type: 1 = Subordinate Only, 2 = Middle Manager. S.R. = Self-Reported; F. R. = Follower (subordinate)-Reported; EI = Emotional Intelligence; EIB = emotionally intelligent behavior. *p < 0.05, **p < 0.01, ***p < 0.001.

Table 2 reports the results for cross-level polynomial regression analyses for subordinate burnout and job engagement. For both burnout and job engagement the polynomial models (Model 2) fit significantly better than did models with only control variables, and the conditional *R*^2^ values increased across these models. Furthermore, the polynomial terms were statistically significant, justifying further response surface analyses.

**Table 2:** Multilevel polynomial regression of supervisor self- and subordinate-reported EIB on subordinate engagement and burnout.

	Subordinate Job Engagement						Subordinate Work-Related Burnout					
	Model 1		Model 2		Model 3		Model 1		Model 2		Model 3	
	*b*	*SE*	*b*	*SE*	*b*	*SE*	*b*	*SE*	*b*	*SE*	*b*	*SE*
Intercept	2.557***	0.443	2.813***	0.444	2.846***	0.444	5.706***	0.908	4.965***	0.863	5.400***	0.866
Controls												
Hospital dummy 1	0.086	0.060	0.112^†^	0.062	0.131*	0.059	0.197	0.159	0.120	0.150	0.133	0.146
Hospital dummy 2	0.047	0.428	0.032	0.429	0.148	0.430	0.980	0.875	1.023	0.831	1.323	0.838
Hospital dummy 3	0.166	0.423	0.190	0.422	0.253	0.423	1.275	0.862	1.110	0.818	1.510^†^	0.823
Hospital dummy 4	−0.160***	0.044	−0.147**	0.045	−0.161***	0.044	0.104	0.110	0.060	0.104	0.071	0.101
Supervisor sex	−0.027	0.034	−0.027	0.035	−0.015	0.034	−0.068	0.089	−0.062	0.084	−0.054	0.082
Supervisor age (years)	0.008***	0.002	0.007***	0.002	0.008***	0.002	−0.006	0.005	−0.007	0.004	−0.009*	0.004
Supervisor tenure (years)	−0.004*	0.002	−0.004*	0.002	−0.004*	0.002	−0.003	0.004	−0.003	0.004	−0.003	0.004
Supervisor type	−0.220	0.417	−0.228	0.416	−0.287	0.417	−1.228	0.844	−1.197	0.801	−1.563^†^	0.806
Subordinate gender	−0.046	0.032	−0.049	0.032	−0.048	0.032	0.005	0.062	0.002	0.059	0.021	0.059
Subordinate age (years)	0.007***	0.001	0.007***	0.001	0.007***	0.001	−0.013***	0.003	−0.011***	0.003	−0.011***	0.003
Subordinate tenure (years)	−0.007***	0.002	−0.006***	0.002	−0.006***	0.002	0.021***	0.003	0.016***	0.003	0.016***	0.003
Subordinate type	0.162***	0.046	0.122**	0.047	0.132**	0.046	0.125	0.096	0.268**	0.091	0.249**	0.091
Subordinate S.R. EI	0.416***	0.024	0.377***	0.025	0.378***	0.025	−0.105*	0.045	−0.012	0.044	−0.016	0.044
Independent variables												
Supervisor F.R. EIB (x)			0.121***	0.022	0.116***	0.023			−0.324***	0.039	−0.302***	0.041
Supervisor S. R. EIB (y)			−0.049	0.057	−0.069	0.058			0.099	0.132	0.119	0.134
x^2^			0.025**	0.008	0.026***	0.008			−0.015	0.014	−0.015	0.014
x × y			−0.007	0.027	−0.001	0.028			−0.049	0.049	−0.080	0.052
y^2^			0.058	0.054	0.053	0.055			−0.107	0.127	−0.149	0.130
Moderator terms												
Supervisor EI ability (w)					0.004*	0.002					−0.005	0.004
x × w					−0.002	0.001					0.003	0.003
y × w					0.000	0.004					0.015	0.009
x^2^ × w					−0.001	0.000					0.000	0.001
x × y × w					0.003	0.002					−0.006^†^	0.004
y^2^ × w					−0.001	0.004					0.002	0.009
Deviance	3502.3		3449.0		3434.9		6025.6		5789.5		5777.7	
*df*	2015		2010		2004		2014		2009		2003	
Δχ^2^			53.304***		14.104*				236.093***		11.771^†^	
*R* ^2^_conditional_	18.84%		22.03%		21.79%		15.28%		25.48%		25.53%	

Supervisor *N* = 202 for Job Engagement and *N* = 201 for Burnout; Subordinate *N* = 2,031 for Job Engagement and *N* = 2,030 for Burnout. Gender: 1 = Women, 2 = Men; Supervisor Type: 1 = Middle Manager (i.e., both a subordinate and supervisor), 2 = Senior Manager; Subordinate Type: 1 = Subordinate Only, 2 = Middle Manager. S.R. = Self-Reported; F.R. = Follower (Subordinate)-Reported. EI = Emotional Intelligence; EIB = Emotionally Intelligent Behavior. ^†^*p* < 0.10, *p < 0.05, **p < 0.01, ***p < 0.001.

**Figure 3:**
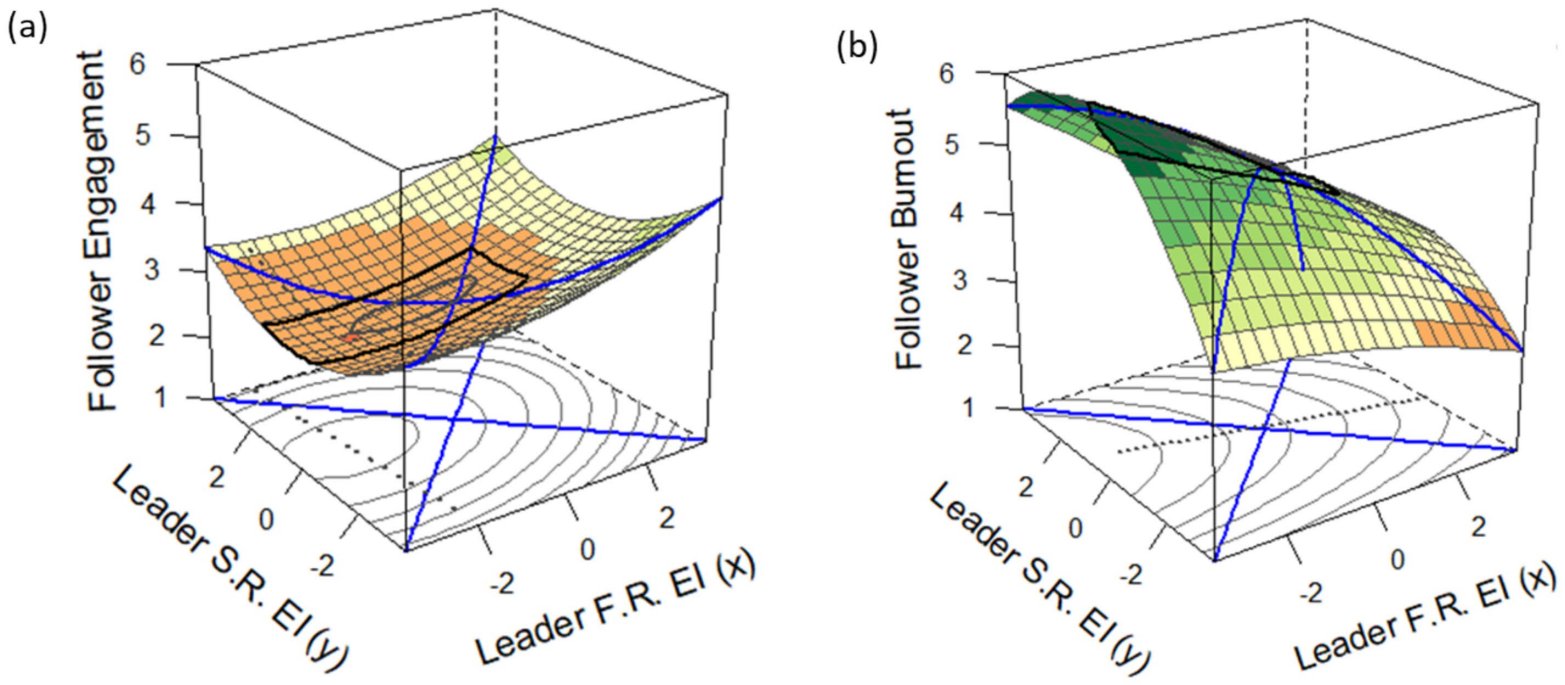
Surface plots depicting the joint effects of supervisor self-reported and subordinate-reported EIB on follower **(a)** job engagement and **(b)** burnout.

Table 3 contains the surface values and the principal axes for both outcomes, of which the second principal axes of incongruence (*p*_20,_ *p*_21_) related to burnout and job engagement and Figure 3 contains visual plots of the response surfaces. Supporting the congruence hypothesis (H1), the analyses showed that congruence in supervisor-subordinate perceptions of supervisor EIB was associated with lower subordinate burnout (H1a) and higher engagement (H1b). Testing for difference between congruence on high versus low supervisor EIB, for burnout, the slope of the line of congruence was nonsignificant, *b* = −0.255, *p* = 0.083, and the confidence intervals for *p*_10_ included zero and for *p*_11_ included one; similar patterns were observed for the secondary principal axes. For engagement, the confidence intervals for *p*_10_ and *p*_11_ also encompassed zero and one, respectively, and again this pattern held for the secondary axes. These results indicate that the surfaces did not show contingency effects, and the slopes of the congruence lines for both burnout and engagement were nonsignificant (burnout: *b* = −0.255, *p* = 0.083; engagement: *b* = 0.072, *p* = 0.200). Although the first principal axis for engagement (Figure 3) was visually not fully aligned with the congruence line, p10 and p11 showed that this deviation was nonsignificant. Thus, while no statistical differences emerged between the two forms of congruence, both congruence forms showed the same directional pattern—relating negatively to burnout and positively to engagement, supporting H1a and H1b.

**Table 3:** Slopes along lines of congruence and incongruence, and principal axes for supervisor self-rated and supervisor subordinate-rated EIB on subordinate job engagement and work-related burnout.

	Job engagement	Burnout
Surface values		
*a*_1_: Line of Congruence (x = y), Slope (x + y)	0.072 (0.056)	−0.225 (0.130)^†^
*a*_2_: Line of Congruence (x = y), Curvature (x^2^ + x*y + y^2^)	0.076 (0.054)	−0.170 (0.126)
*a*_3_: Line of Incongruence (x = −y), Slope (x – y)	0.169 (0.066)*	−0.423 (0.145)**
*a*_4_: Line of Incongruence (x = −y), Curvature (x^2^ – x*y + y^2^)	0.090 (0.067)	−0.073 (0.146)
Principal axes		
*p*_10_: First Principal Axis, Intercept	−21.110	0.083
*p*_11_: First Principal Axis, Slope	−9.034	−0.247*
*p*_20_: Second Principal Axis, Intercept	0.533	81.877
*p*_21_: Second Principal Axis, Slope	0.111	4.042

x = Supervisor Subordinate-Reported EIB; y = Supervisor Self-Reported EIB. Coefficients are outside of the parentheses and SEs are inside. For intercepts and slopes of the first and second principal axes, an asterisk (*) indicates the confidence intervals exclude zero at p < 0.05 based on the values from the Monte Carlo simulations. ^†^*p* < 0.10, *p < 0.05, **p < 0.01, ***p < 0.001.

There are two important considerations for interpreting response surfaces. First, it is important to evaluate all response surface parameters simultaneously within a single model (Yao and Ma, 2022). Thus, the results were examined for each outcome separately in relation to the hypotheses. Following recommendations from Edwards and Cable (2009), the intercept (*p10*) and slope (*p11*) of the first principal axis (i.e., congruence) and the intercept (*p20*) and slope (*p21*) of the second principal axis (i.e., incongruence) were examined. The surface values were interpreted as recommended by Yao and Ma (2022; see their Table 4).

Second, when examining the response surface plots, it is appealing to focus on the corners because upward or downward bends are more pronounced (Schönbrodt, 2016). However, the corners are often areas in which there are no actual observations, and the slopes/curvatures are assumed. Thus, it is recommended to only interpret the regions in which there is actual data. To aid this interpretation, the range of centered supervisor subordinate-reported EIB scores was −3.59 to 1.41 and the range for centered supervisor self-reported EIB scores was −1.23 to 1.41. This is graphically displayed in Figure 3 based on the black ovals on the surfaces. Further, the dotted line in the graph is the principal axis.

In examining the incongruence hypothesis (H2), for subordinate burnout, the confidence intervals for the intercept and slope of the second principal axis excluded zero and one, respectively. This aligned with the interpretation of the surface values as well, as the slope of the line of incongruence was significant, *b* = −0.423, *p* = 0.003. Put simply, subordinate burnout was significantly higher when supervisors overestimated their EIB compared to when supervisors underestimated their EIB. Hence, H2a was supported. H2b was also supported, as the slope of the line of incongruence for subordinate job engagement was significant, b = 0.169, *p* = 0.010. Put simply, subordinate engagement was significantly lower for subordinates of supervisors who overestimated their EIB compared to subordinates whose supervisors underestimated their EIB.

Finally, the incongruence effect on burnout and engagement remained significant after supervisor EI ability was controlled for (Model 3), suggesting that the differential effects of supervisor underestimation and overestimation are not attributed to EI ability.

## Discussion

This study examined how (mis)alignment between supervisor and subordinate ratings of supervisor EIB relates to subordinate the well-being indicators burnout and engagement. Three key findings emerged. First, supervisor-subordinate perceptual congruence on supervisor EIB was positively associated with subordinate engagement and negatively associated with subordinate burnout, independent of the perceived level of supervisor EIB. Second, when incongruence occurred, subordinates reported the lowest engagement and highest burnout when supervisors overestimated their EIB relative to subordinates’ ratings, compared to cases of supervisor underestimation. Third, these patterns could not be explained by supervisors’ EI ability.

### Theoretical implications

This study offers several theoretical contributions by applying and extending Self–Other Agreement theory (Atwater and Yammarino, 1992, 1997) and the Job Demands–Resources model (Bakker and Demerouti, 2017, 2024) to the context of EI in supervisor–subordinate relationships. Whereas prior research tended to examine supervisors’ EI ability, subordinates’ perceptions of supervisor EIB, or supervisors’ perceptions of their EIB in isolation, our study shifts the focus toward the relational nature of EI — specifically, the (mis)alignment between supervisors’ and subordinates’ perceptions of supervisor EI-relevant behavior—and demonstrates its relevance to subordinate well-being. The present study does not focus on supervisor individual attributes (whether self-perceived or attributed by others), but instead focuses on perceptions of enacted behavior, which is a more proximal influence on subordinate outcomes (compared to internal attributes; Ivcevic et al., 2025; Levitats et al., 2025).

A key contribution of this study concerns the role of perceptual (mis)alignment in shaping subordinate well-being. We foreground the relational nature of EIB, demonstrating that (mis)alignment between supervisors’ and subordinates’ perceptions has important implications for subordinate engagement and burnout. Consistent with SOA theory, we show that alignment and misalignment in perceptions have distinct and opposing associations with subordinate well-being, regardless of supervisors’ actual EI ability. Our findings suggest that a shared understanding of supervisor EIB functions as a psychologically stabilizing force in the workplace and may trigger resource gain spirals (Hobfoll, 2001; Westman et al., 2004), amplifying engagement and protecting against burnout.

These findings resonate with evidence showing that perceptual (in)congruence within workplace relationships meaningfully shapes subordinate well-being. In a study of perceptual (in)congruence on qualitative job insecurity between individuals and their teammates, Roczniewska and Richter (2022) demonstrate that alignment, whether at low or high levels, tends to stabilize subordinates’ engagement, whereas perceptual incongruence can undermine both engagement and recovery. Additional evidence comes from research on supervisor-subordinate dyads; for example, Zhao et al. (2024) found that congruence between supervisor and subordinate evaluations of supervisor transformational leadership behavior strengthened the positive effects of subordinate-rated leadership on team performance compared to incongruence, demonstrating that alignment itself creates a beneficial relational condition.

Beyond the general benefits of perceptual alignment, our findings highlight important differences between forms of supervisor–subordinate incongruence in perceptions of supervisor EIB. Prior research conceptualizes supervisors’ low EI-related attributes as job demands and high EI-related attributes as job resources (Ivcevic et al., 2021; Levitats et al., 2019). We find that subordinates with underestimating supervisors showed higher engagement levels and lower burnout levels compared to those working with overestimating supervisors. This pattern supports our prediction drawn from the JD-R model (Bakker and Demerouti, 2017, 2024) that supervisor EIB overestimation creates a particularly demanding work environment. It seems that when supervisors overestimate their EIB, subordinates encounter dual resource depletion: they both have to interact with supervisors who show insufficient recognition, understanding, and regulation of emotions and have the additional burden of regulating their behavior not to indicate to the supervisors that their EIB is ineffective. Although subordinates working under EIB underestimating supervisors are not “in-sync” with their supervisors’ perceptions either, they report higher engagement and lower burnout. A plausible explanation is that underestimating supervisors’ self-critical orientation motivates efforts toward self-improvement (Tekleab et al., 2008), leading them to enact emotionally intelligent behaviors that generate compensatory job resources for subordinates, thereby fostering engagement and buffering against burnout.

Finally, our findings suggest that the meaning of EIB emerges through a relational and contextual lens. By showing that supervisor-subordinate congruence on perceived supervisor EIB has meaningful implications for subordinate burnout and engagement, beyond their EI ability, our findings speak directly to calls to expand EI research toward understanding how EIB is interpreted in practice. It should be noted that these findings do not diminish the relevance of supervisors’ EI ability but rather reinforce the importance of further studying how EIB is socially interpreted within dyadic relationships. EI ability remains a foundational component that represents the potential to reason about emotions. However, it does not automatically translate into behavior (Ivcevic et al., 2025; Levitats et al., 2025; Lopes, 2016).

### Practical implications

Our findings offer several practical implications for organizations seeking to enhance subordinate well-being. First, since perceptual alignment itself benefits subordinate well-being, organizations should promote open dialogue and feedback mechanisms to foster mutual understanding between supervisors and subordinates about how they act to show perceiving, using, understanding, and regulating emotions. Tools such as 360-degree feedback, structured check-ins, and team reflection sessions may help supervisors reconcile their self-perceptions with subordinates’ experiences of their emotional leadership. Evidence from prior work illustrates this point. For example, Nielsen et al. (2022) integrated SOA theory into leadership training programs and demonstrated that feedback-driven development can improve supervisor-subordinate agreement on the supervisor’s leadership behaviors.

The example above emphasizes how structured feedback can serve as a mechanism for enhancing self–other agreement. Building on this insight, leadership development programs should focus on helping supervisors who overestimate their EIB recognize and address these perceptual gaps by adopting practical behaviors aligned with the four core components of EI (Mayer and Salovey, 1997). Recent theoretical work (Levitats et al., 2025) and work aimed at practitioners (Ivcevic et al., 2025) outlines influences on such behavior and provides guidance for developing this behavior in supervisors. Finally, because perceptual alignment influences subordinate well-being independently of actual EI ability, organizations may benefit from incorporating alignment-focused assessments, such as supervisor–subordinate perceptual gap analyses, into performance evaluations.

### Limitations

This study provides important insights into the relationship between supervisor-subordinate perceptual alignment of supervisor EIB and subordinate well-being. Yet, it has several constraints that suggest directions for further investigation. A central limitation of this study is its cross-sectional design, which restricts our ability to infer causality. Both predictor and outcome measures were administered at the same time, with only supervisor EI ability administered at a different time. Though reverse causation does not seem theoretically meaningful, it remains possible that subordinate well-being also influences how subordinates perceive their supervisor’s EI. Future research could build on this work and employ longitudinal or time-wave approaches, to help disentangle the temporal ordering of these processes by tracking changes in perceptual alignment and well-being across time. Repeated measures would also allow researchers to capture *dynamic alignment patterns*, such as whether recurrent misalignment is more harmful than temporary discrepancies.

A second limitation concerns the contextual specificity of the study. The hospital environment provides a unique setting for examining EI alignment, yet its generalizability may be limited. Healthcare organizations are characterized by strong professional cultures, hierarchical structures, and highly emotional work, all of which distinguish them from many other organizational contexts. While the observed patterns may extend to other high-stakes or service-oriented environments, further research in non-clinical settings is needed to determine whether the effects of EI alignment manifest differently.

## Conclusion

This study highlights the role of alignment between supervisors and subordinates perceptions of supervisor EIB. By focusing on perceptual (mis)alignment, we offer a partner and socially grounded understanding of EI in the workplace. Our findings show that alignment, regardless of perceived EIB level, is associated with higher engagement and lower burnout, suggesting that shared understanding itself serves as a psychological resource. Importantly, when supervisors overestimate their EIB relative to subordinates’ perceptions, subordinate well-being suffers most, underscoring the emotional cost of misalignment. These effects are independent of the supervisor’s EI ability, suggesting that subordinate outcomes are not associated with just supervisor emotion-related know how, but also the match or mismatch in how supervisors and subordinates perceive supervisor behavior.

## Data Availability

The raw data supporting the conclusions of this article will be made available by the authors, without undue reservation.
