# Supplementary material for: Beyond emotional intelligence ability: the power of supervisor–subordinate alignment in perceptions of supervisor emotionally intelligent behavior
Source: Front Psychol. 2026 Mar 3;17:1637168. doi: 10.3389/fpsyg.2026.1637168 (PMC12991986; doi:10.3389/fpsyg.2026.1637168)
Supplement: Supplementary file 1 [file Data_Sheet_1.docx]

**APPENDIX 1**

For polynomial regression, there are various considerations for test of statistical significance (Edwards & Parry, 1993). First, equations involving products and ratios of the regression coefficients (i.e., principal axes and slopes along the principle axes) cannot be calculated using traditional approaches as equations for calculating standard errors are often unavailable. Edwards and Parry (1993; see also Edwards & Cable, 2009) recommend conducting nonparametric bootstrapping to calculate confidence intervals for such tests. However, this is not possible with multilevel data because of the clustered sampling (Zyphur et al., 2016). Thus, following recommendations from Zyphur et al. (2016), a Monte Carlo approach was used to simulate estimates for the parameters that could then be used to conduct statistical significance tests. For each of the four outcomes, the observed parameter estimates (*x*, *y*, *x*2, *x* × *y*, *y*2), the parameter variances, and the covariances were used in a Monte Carlo simulation to generate 10,000 estimates that were then used to compute 10,000 response surfaces. The distribution of these response surfaces was used to determine if the response surface terms were statistically significant based on percentile ranking (i.e., the bottom 2.5% and top 97.5% for each response surface parameter).

Beyond the above considerations, there are two important pre-requisites for polynomial regression and response surface analyses. First, for polynomial regression, it is ideal to show there is adequate (in)congruence in the sample (Humberg et al., 2020; Wilson et al., 2020). For example, if all supervisors and subordinates agreed or disagreed it would be difficult to accurately test any (in)congruence relationships. Following Humberg and colleagues' recommendations, it was examined in which percentage of the sample each type of supervisor- subordinate agreement about the supervisor’s EI occurred in which percentage occurred each type of disagreement (i.e., (a) overestimation: supervisors rate themselves higher than do their subordinates and (b) underestimation: supervisors rate themselves lower than do their subordinates). For this analysis, the combined grand mean of both EI variables was used, to ensure that the proportion of (in)congruent scores are based on the same scaling for both variables (i.e., commensurability). Scores were considered congruent if they were within ±0.5*SD* and incongruent if outside of that range. Overall, 35 percent of supervisor-subordinate rating pairs were congruent, 54 percent of supervisor-Subordinate pairs were incongruent such that supervisor’s overestimated their scores, and 11 percent of scores were incongruent such that supervisor’s underestimated their scores. Although there are no norms or cutoffs for what proportion is reasonable, this seems to be a good distribution of scores.

The second pre-requisite applies to response surface methodology. Before conducting these analyses, some of the five polynomial regression terms should be statistically significant (Edwards, 1996). Previously, there have been different views for how to evaluate the significance of these terms, creating ambiguity (Yao & Ma, 2022). Some studies tested the incremental effect of the three higher order polynomial terms (e.g., Jansen et al., 2016), whereas others tested the statistical significance of all five terms simultaneously (King et al., 2017). Recently, Yao and Ma (2022) noted that researchers should focus on all five terms as it is still possible to find significant congruence effects even when the three higher order terms are nonsignificant. Thus, incremental significance for the entire block of five terms was examined – based on changes in model deviance scores (-2 log likelihood) from a model with control variables only. Additionally, *R*^2^ values are reported for tests of practical significance of each model. These values were calculated using the MuMIn R package v1.43.17 (Bartoń, 2020). Because *R*^2^ values are difficult to calculate for multi-level analyses, conditional *R*^2^ values were relied upon, as suggested by Nakagawa et al. (2017). Conditional *R*^2^ values reflect the proportion of variance explained by the fixed and random effects over total variance (fixed effects, random effects, and model residuals).
